# Osteoblast-Derived Paracrine and Juxtacrine Signals Protect Disseminated Breast Cancer Cells from Stress

**DOI:** 10.3390/cancers13061366

**Published:** 2021-03-18

**Authors:** Russell Hughes, Xinyue Chen, Natasha Cowley, Penelope D. Ottewell, Rhoda J. Hawkins, Keith D. Hunter, Jamie K. Hobbs, Nicola J. Brown, Ingunn Holen

**Affiliations:** 1 Department of Oncology and Metabolism, University of Sheffield, and Experimental Cancer Medicine Centre, Sheffield S10 2RX, UK; xinyue.chen@sheffield.ac.uk (X.C.); p.d.ottewell@sheffield.ac.uk (P.D.O.); n.j.brown@sheffield.ac.uk (N.J.B.); i.holen@sheffield.ac.uk (I.H.); 2 Department of Physics and Astronomy, University of Sheffield, Sheffield S3 7RH, UK; natasha.cowley@sheffield.ac.uk (N.C.); rhoda.hawkins@physics.org (R.J.H.); jamie.hobbs@sheffield.ac.uk (J.K.H.); 3 School of Clinical Dentistry, University of Sheffield, Sheffield S10 2TA, UK; k.hunter@sheffield.ac.uk

**Keywords:** skeletal, metastasis, dormancy, latency, breast cancer

## Abstract

**Simple Summary:**

Bone metastasis is a debilitating, incurable complication that occurs in breast cancer patients with a variable period of latency after treatment of the primary tumor. The seeding of individual cancer cells in the bone marrow from the primary tumor occurs early in disease progression. How disseminated cancer cells are able to remain dormant in the bone marrow and then reactivate decades later to form destructive bone lesions is not fully understood. We have used model systems to identify bone resident cell types, and molecular mediators, that influence the survival and fate of disseminated breast cancer cells. The identification of such cellular and molecular support mechanisms can inform the design of new therapeutic strategies aimed at eliminating indolent cancer cells from the bone marrow, preventing progression to overt metastases.

**Abstract:**

Metastatic breast cancer in bone is incurable and there is an urgent need to develop new therapeutic approaches to improve survival. Key to this is understanding the mechanisms governing cancer cell survival and growth in bone, which involves interplay between malignant and accessory cell types. Here, we performed a cellular and molecular comparison of the bone microenvironment in mouse models representing either metastatic indolence or growth, to identify mechanisms regulating cancer cell survival and fate. In vivo, we show that regardless of their fate, breast cancer cells in bone occupy niches rich in osteoblastic cells. As the number of osteoblasts in bone declines, so does the ability to sustain large numbers of breast cancer cells and support metastatic outgrowth. In vitro, osteoblasts protected breast cancer cells from death induced by cell stress and signaling via gap junctions was found to provide important juxtacrine protective mechanisms between osteoblasts and both MDA-MB-231 (TNBC) and MCF7 (ER^+^) breast cancer cells. Combined with mathematical modelling, these findings indicate that the fate of DTCs is not controlled through the association with specific vessel subtypes. Instead, numbers of osteoblasts dictate availability of protective niches which breast cancer cells can colonize prior to stimulation of metastatic outgrowth.

## 1. Introduction

The spread of cancer cells to the bone marrow occurs frequently during breast cancer progression [1,2] and significantly increases the risk of skeletal recurrence [2]. However, only a proportion of patients with tumor cells in their bone marrow will subsequently develop clinically overt metastases, often following a prolonged period of disease latency [1,2]. The mechanisms that influence the survival and fate of disseminated tumor cells (DTCs) in bone during this period of metastatic dormancy are not fully understood but remain an attractive therapeutic target.

Following their dissemination to bone, tumor cells are proposed to reside in hematopoietic stem cell (HSC) niches located in proximity to blood vessels (perivascular), bone surfaces (endosteal) or both (overlapping) [3,4,5]. Therefore, the fate of DTCs in bone is likely influenced by their interactions with the cell types that co-exist in these niches. The microvasculature in bone is complex and plays an important part in the homeostatic regulation of the HSC pool, including control of their egress into the blood stream and subsequent re-entry into bone marrow niches [6]. Circulating tumor cells (CTCs) are proposed to hijack such HSC homeostatic processes, thereby gaining entry to the bone microenvironment [3] and the perivascular niche has also been implicated as an important regulator of metastatic dormancy in bone [7,8,9]. Broadly, the bone microvasculature can be divided into two functionally distinct vessel subtypes. Blood vessels that co-express high levels of the vascular markers CD31 and endomucin (termed type-H vessels) are more proliferative than those that express low or undetectable levels of CD31 (termed type-L vessels). Forming an important functional link between blood vessel and bone formation, H-type^CD31pos^ vessels have a transcriptional profile enriched for osteogenic growth factors and can regulate the expansion and differentiation of bone forming cells [10]. Osteoblasts, derived from mesenchymal stromal/stem cells (MSCs), are the key cell type responsible for bone formation. During their differentiation in bone, these cells can reside in both perivascular and endosteal niches, where they regulate HSC activity [11,12]. Consequently, osteoblastic cells are implicated as one of the key accessory cell types to DTCs during the early stages of tumor cell colonization of bone [13]. However, the specific roles of osteoblastic cells and different types of vessels in controlling the survival of DTCs in bone during metastatic dormancy and outgrowth are not fully understood.

The metastatic cascade is highly inefficient, with the majority of malignant cells (>90%) unable to survive the stresses associated with the multistep metastatic process [14,15,16]. Successful colonization of metastatic sites likely relies on DTCs locating to appropriate niches, with the recruitment of accessory cell types to support their long-term survival. The aim of this study was to identify cellular and molecular mechanisms that regulate the fate of DTCs in bone by comparing the bone microenvironment from models with opposing effects on the fate of DTCs, one driving outgrowth and the other promoting indolence. Using this approach, we determined that DTCs reside in microanatomical niches populated by osteoblastic cells. As the abundance of osteoblastic cells declines in our indolence-inducing mouse models, so does the ability of the bone microenvironment to sustain large numbers of DTCs. Using in vitro co-culture assays, we demonstrated that osteoblasts deploy both paracrine and juxtacrine signals that increase tumor cell resistance to metastasis-induced cell stress and death. We propose that osteoblasts provide a limited number of niches capable of supporting the long-term survival of DTCs in bone and that the innate ability to grow and form metastases is restricted to a small percentage of DTC clones. By limiting the numbers of sites for colonization by DTCs, osteoblasts indirectly limit the likelihood of metastatic outgrowth.

## 2. Results

### 2.1. Disseminated Breast Cancer Cells Occupy Distinct Micro-Anatomical Niches during Metastatic Dormancy Compared to Outgrowth

**Figure 1:**
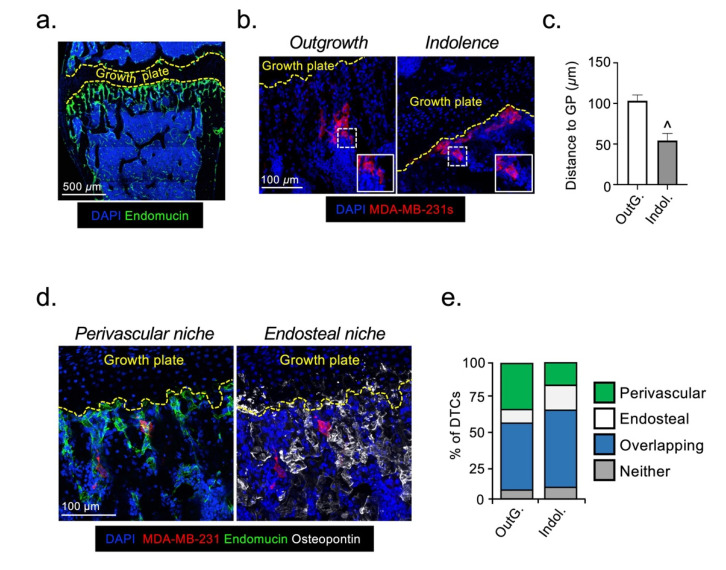
DTCs occupy distinct micro-anatomical niches in bone during metastatic indolence and outgrowth. (**a**) Illustrative immunofluorescent image of the metastatic region of mouse tibial metaphysis. The growth plate is indicated by yellow dashed line and the endomucin expressing microvasculature in green. (**b**) Representative images of MDA-MB-231 DTCs in tibial cryo-sections taken from the outgrowth and indolence mouse models, acquired 6 days post-injection. Inset panel shows high power image of individual tumor cells (in red). (**c**) Quantitative mapping of DTC distance to the growth plate in outgrowth and indolence models. (**d**) Representative images of MDA-MB-231 DTCs (in red) residing in perivascular (in green) and endosteal (in white) niches, in vivo. (**e**) Graphical summary and comparison of DTC distribution between perivascular and endosteal niches in the outgrowth and indolence mouse models. (OutG. = Outgrowth, Indol. = Indolence; ^ sig. dif. w.r.t. outgrowth (*p* < 0.0001); *n* = 5 mice/tibia per condition).

To determine how the bone microenvironment influences the survival and fate of DTCs, we used two mouse models that differ in their ability to support cancer cell growth following their arrival in bone after intra-cardiac injection [17,18]. In the first model (6-week-old, skeleton still developing), cancer cells rapidly grow to form overt bone metastases. In the second model (12-week-old, mature skeleton with lower bone turnover), cancer cells remain indolent for an extensive period of time until reactivated [17]. Regardless of molecular subtype, our group has previously demonstrated that breast cancer cells labelled with fluorescent, lipophilic membrane dyes preferentially colonize the metaphyseal bone compartment in mouse models [19]. To determine whether the distribution of DTCs (MDA-MB-231) in this bone region might influence their fate, we mapped their distance relative to the growth plate, a readily identifiable anatomical feature, shortly after long bone colonization in the mouse models of outgrowth and indolence. This was carried out via a more precise method using the immunofluorescent detection of endogenous tumor cell antigens (CD29 and CD59), which unlike lipophilic membranes dyes, is not subject to lateral dye transfer. In both models, DTCs were detected in niches located within 200 µm from the growth plate (Figure 1a,b), demonstrating that the differential fate of DTCs cannot be explained by macroscopic differences in their distribution. However, in the indolent model there was a statistically significant micro-anatomical shift in tumor cell distribution, with DTCs located significantly closer to the growth plate compared with the outgrowth model (Figure 1c).

The perivascular and endosteal niches are strongly implicated in the regulation of HSC proliferation and dormancy [12,20,21,22,23,24], and there is compelling evidence that DTCs hijack HSC niches in bone [3,4,5,9]. We hypothesized that the shift in DTC location observed in the indolence model could reflect either a reconfiguring of these niches, or transition of DTCs between them. To investigate this, we established the extent to which DTCs associated only with either the perivascular niche (using the vascular marker endomucin) [10], the endosteal niche (using the surrogate bone marker osteopontin) [25], or with both. In both outgrowth and indolent models, ~50–60% of the DTCs were associated with an overlapping niche that expressed both perivascular and endosteal markers (Figure 1d). Importantly, there was a degree of redistribution of DTCs between different locations in the two models. In the indolent model, there was a statistically significant reduction in the proportion of DTCs associated with only the perivascular niche (16.1% ± 4.1), compared with the outgrowth model (33.4% ± 5.0; *p* < 0.01). There was a corresponding increase in DTC association with only the endosteal niche (dormancy 18.3% vs. outgrowth 10.0%; *p* < 0.05). These data reveal a micro-anatomical repositioning of a proportion of DTCs in bone, between the perivascular and endosteal niches during the transition from metastatic indolence to outgrowth in vivo. Our results demonstrate that changes in both perivascular and endosteal niches may affect the growth or survival of DTCs in bone.

### 2.2. The Fate of DTCs in Bone Is Not Determined by Their Interaction with Specific Vessel Subtypes

**Figure 2:**
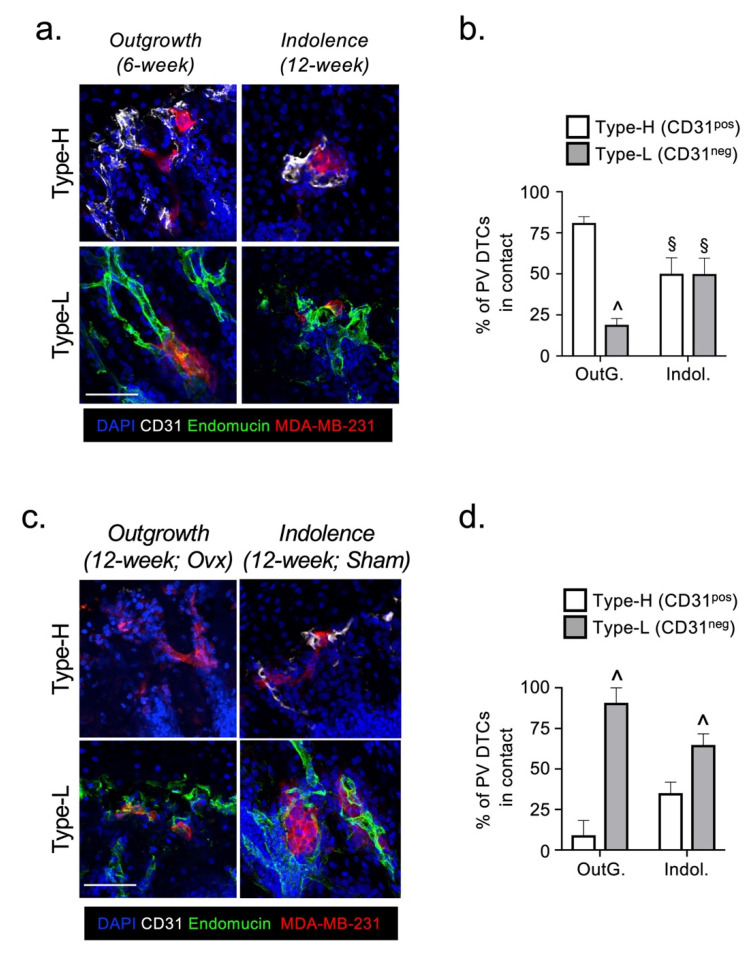
The fate of DTCs in bone is not determined by their association with osteogenic vascular endothelium. Representative immunofluorescent images (**a**) and quantification (**b**) of MDA-MB-231 DTCs (in red) associated with CD31^pos^ type-H (in white) and CD31^neg^ type-L (in green) microvasculature in the bones of 6-week old (Outgrowth model) and 12-week old (Indolence model) mice. Representative images (**c**) and quantification (**d**) of MDA-MB-231 DTCs associated with CD31^pos^ type-H and CD31^neg^ type-L microvasculature in the bones of ovariectomised 12-week old mice (Outgrowth) and sham-operated 12-week old mice (Indolence). (OutG. = Outgrowth, Indol. = Indolence; ^ sig. dif. w.r.t. CD31^+^ (*p* < 0.01), § sig. dif. w.r.t. Outgrowth (*p* < 0.01); *n* = 3 mice/tibia per condition).

We next investigated how the repositioning of DTCs between the perivascular and endosteal niches in bone might influence their survival and ability to form overt metastatic lesions. We and others have previously reported a decline in the abundance of type-H^CD31pos^ blood vessels in mature mice [10,26]. Thus, if CD31^pos^ endothelial cells (ECs) provide proliferative cues to DTCs, a reduction in this EC population could explain the absence of metastatic outgrowth in our model of indolence. To explore this possibility, we quantified DTC association with either type-H^CD31pos^ or type-L^CD31neg^ vessels in the two models. In the outgrowth model, perivascular DTCs showed a statistically significant bias towards type-H blood vessels (Figure 2a,b). In the indolence model, DTCs showed no bias with an equivalent association with either vessel type (Figure 2a,b). These data were consistent with type-H^CD31pos^ ECs supporting the proliferation of DTCs. To validate these findings, we used a mouse model in which indolent DTCs in the bone marrow of mature mice are triggered to form overt metastatic lesions following ovariectomy, but not following a sham operation [17]. However, in ovariectomized mice (outgrowth model), perivascular DTCs showed a statistically significant bias towards type-L blood vessels, which was also present in the sham-operated mice (indolence model) (Figure 2c,d). Taken together, these data indicate that the fate of DTCs is not determined by the type of vessel with which they are associated, since outgrowth occurs spontaneously in young mice when DTCs are associated with type-H^CD31pos^ vessels and in ovariectomized mature mice whilst associated with type-L^CD31neg^ vessels.

### 2.3. Osteoprogenitors Are Potential Accessory Cells for DTCs

**Figure 3:**
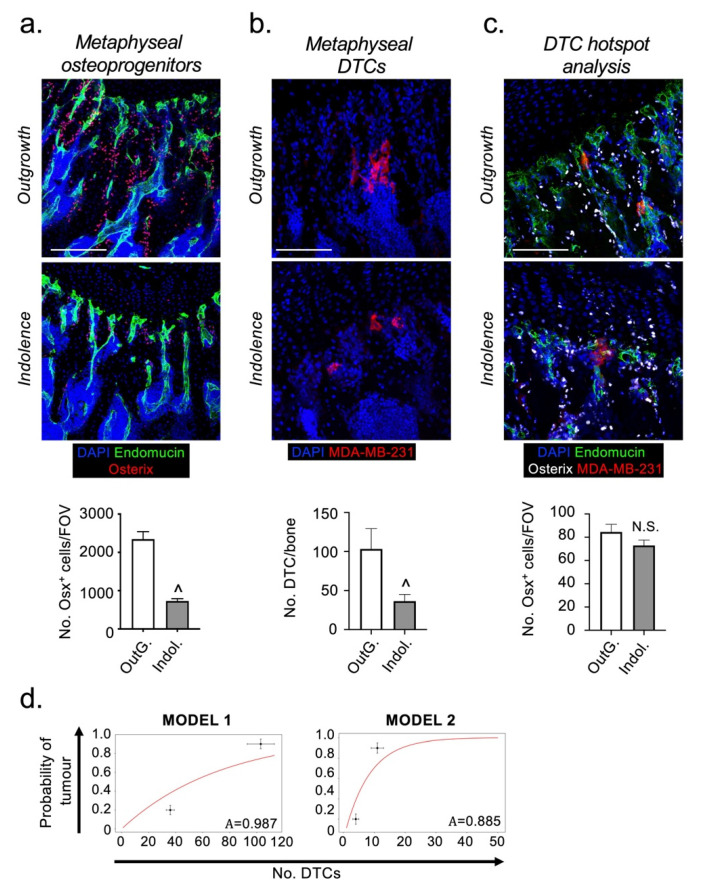
DTCs reside in bone microenvironments rich in osterix-expressing osteoblastic cells in both metastatic outgrowth and indolence models. (**a**) Representative immunofluorescent images and quantification of Osterix^+^ osteoblastic cells (in red) in tibias taken from the outgrowth and indolence models (vasculature shown in green). (**b**) Representative immunofluorescent images and quantification of DTC abundance (in red) in outgrowth and indolence models. (**c**) Representative images of DTCs (in red) residing in micro-anatomical niches containing osterix^+^ osteoblastic cells (in white) and quantification of osterix^+^ osteoblastic cells in FOV containing DTCs “hotspots”. (**d**) Mathematical calculation of the probability of developing tumor growth if all DTCs have an equal likelihood of death vs. proliferation (Model 1) and if proliferation is restricted to a subpopulation (11%) of DTCs (Model 2). Horizontal error bars are standard error of the mean and vertical error bars are based on the accuracy of published data. (OutG. = Outgrowth, Indol. = Indolence; ^ sig. dif. w.r.t. outgrowth (*p* < 0.05), N.S. = not significant; *n* = 5 mice per condition; scale bar = 100 µm).

Osteoprogenitors are found in both perivascular and endosteal niches in bone and may be involved in regulating DTC survival and proliferation/dormancy. Derived from MSCs, osteoprogenitors are the precursors of bone synthesizing osteoblasts and can be identified by their expression of the osteogenic transcription factor ”Osterix” [10]. We quantified the numbers of osteoprogenitors in the metaphyses of hind limbs from the indolence and outgrowth models. Consistent with previously published data [26], osteoprogenitor numbers were significantly reduced in the indolence model (Figure 3a). Interestingly, the reduced abundance of osteoprogenitors was associated with a reduced ability of the bone microenvironment to support high numbers of DTCs, a 65% decrease compared with the outgrowth model (Figure 3b). We hypothesized that osteoprogenitors may be a source of important survival/proliferative cues for DTCs. To test this hypothesis, we quantified the numbers of osteoprogenitors in close proximity to DTCs in the two models. Despite the overall decline in the abundance of osteoprogenitors in the bones of the indolence model (mature mice), DTCs still appeared to reside in micro-anatomical niches containing equivalent numbers of osteoprogenitors in both models (Figure 3c). These data suggest that osteoprogenitors could be an accessory cell that promotes the survival of DTCs in bone, but do not directly affect their ability to remain indolent.

To test the hypothesis that the decline in the number of DTCs in the bones of mature mice at these very early stages after colonization is sufficient to explain the observed metastatic latency, we calculated an expression for the probability of outgrowth, *P(tumor)*, as a function of the number, *n*, of DTCs that were initially observed in bone niches (see methods and Figure S2 for details). Our expression has the form *P(tumor)* = 1 − A^n^, where A is the ratio of the death rate to the proliferation rate of the DTCs after colonization. As we do not have an experimental measurement of these rates, we obtained a value for this parameter by fitting to the established outgrowth data for our mouse models [17]. Initially, we made the assumption that all observed DTCs in both models have the same potential to proliferate and therefore A is equivalent for all DTCs. Our results give a value of A close to 1 i.e., the proliferation rate is only slightly larger than the death rate (Figure 3d). For the case where A ≥ 1, all DTCs eventually die, irrespective of the initial number surviving in bone, which is not seen in our models [17]. However, Kang et al., (2003) [27] and Lu et al., (2011) [28], demonstrated that the ability of MDA-MB-231 cells to grow and form overt metastases in the bones of mice was an intrinsic property restricted to only a subset of TCs (~11%). Therefore, we amended our mathematical model so that only 11% of the DTCs were able to proliferate, this resulted in a fit of A *=* 0.885 (Figure 3d). Using this 11%, the proliferative DTC subpopulation more clearly shows the stochastic effect of small numbers of DTCs, since the fate of each metastatic focus is largely dependent on the initial number of cells surviving after colonization. Based on these assumptions, our calculations suggest that the difference in the number of surviving DTCs in the bones of our two mouse models, is a significant factor in determining their subsequent fate, which is likely influenced directly by the bone microenvironment. However, as can be seen from our graphical data, the theoretical curves do not fully reproduce the observed difference in DTC fate. This suggests the likelihood of additional cellular and molecular interactions within the surrounding bone microenvironment having a minor influence on the death/proliferation rates of DTCs.

### 2.4. Osteoblast-Derived Paracrine Signals Protect Breast Cancer Cells from Oxidative Stress

**Figure 4:**
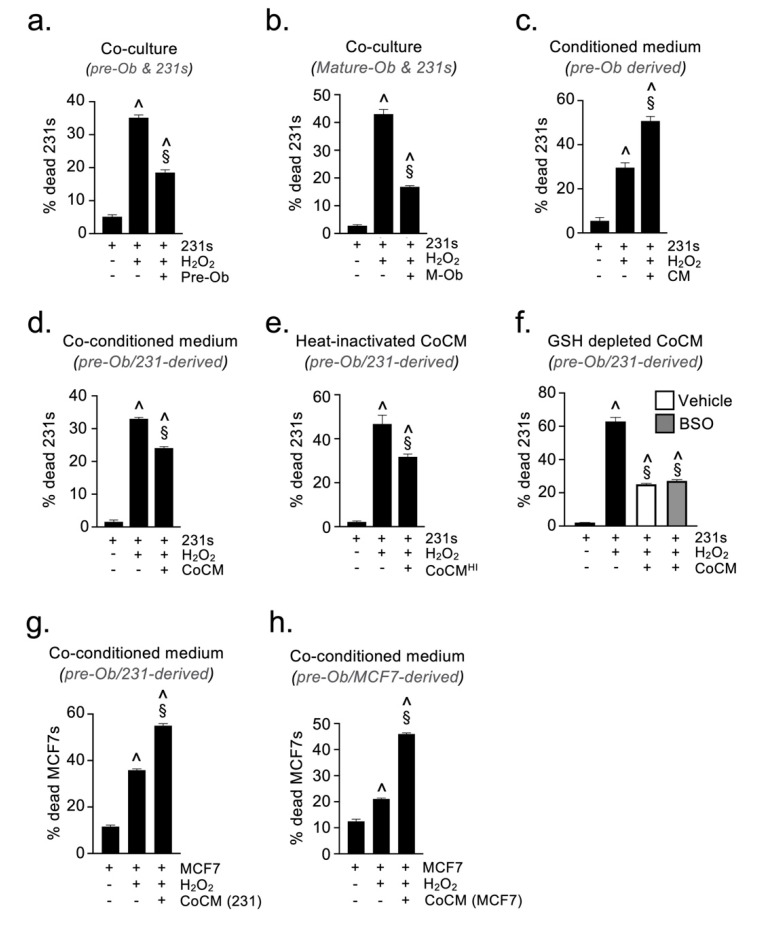
Paracrine cross talk with osteoblasts protects hormone receptor negative BCCs from oxidative stress-induced cell death. Flow cytometric quantification of tumor cell viability in oxidative stress assays performed using MDA-MB-231 BCCs in the presence of (**a**) pre-osteoblasts, (**b**) mature osteoblasts (**c**) pre-osteoblast conditioned medium, (**d**) pre-osteoblast and MDA-MB-231 co-conditioned medium, (**e**) heat-inactivated co-conditioned medium, (**f**) glutathione-depleted co-conditioned medium. Oxidative stress assays performed using MCF7 BCCs in the presence of (**g**) pre-Ob and MDA-MB-231 co-conditioned medium and (**h**) pre-Ob and MCF7 co-conditioned medium. (CM = conditioned medium, CoCM = pre-Ob and BCC co-conditioned medium, HI-CoCM = heat-inactivated pre-Ob and BCC co-conditioned medium, CoCM(231) = co-conditioned medium from MDA-MB-231, CoCM(MCF7) = co-conditioned medium from MCF7, M-Ob = mature osteoblasts; ^ sig. dif. w.r.t. untreated BCCs (*p* < 0.0001), § sig. dif. w.r.t. BCCs + H_2_O_2_ (*p* < 0.01); *n* = 4).

To investigate potential mechanisms whereby osteoprogenitors may influence the survival of DTCs, we established a co-culture assay where MDA-MB-231 breast cancer cells (BCCs) were cultured in the presence of murine MC3T3-E1 pre-osteoblastic cells (pre-Obs). Co-cultures were then analyzed by flow cytometry using the expression of GFP and the cell surface antigens CD29 and CD59 to discriminate between BCCs (GFP^+^CD29/CD59^+^) and pre-Obs (GFP^neg^CD29/CD59^neg^). Co-culture of pre-Obs with BCCs did not significantly alter the viability of either cell type, compared to monocultures under full (10%) serum conditions (data not shown). We hypothesized that osteoprogenitors might protect DTCs from the stress they encountered during the various steps of the metastatic cascade [29,30,31,32], thereby supporting their survival. To test this, co-cultures were performed in the presence of hydrogen peroxide (H_2_O_2_) to simulate oxidative stress. The presence of pre-Obs provided BCCs with significant protection against oxidative stress, reducing the proportion of dead/dying BCCs (Figure 4a). The same protective effect was observed when BCCs were co-cultured with mature osteoblasts under oxidative stress, supporting that this response is independent of osteoblast differentiation status (Figure 4b). To determine whether this protective effect was mediated by paracrine or juxtacrine signals, we repeated the oxidative stress assay with BCCs cultured either alone or in medium conditioned by pre-Ob (CM). Surprisingly, the pre-Ob CM did not protect BCCs against oxidative stress, but significantly increased the proportion of dead/dying BCCs (Figure 4c). These data indicate that the protective effect observed in co-culture experiments must be mediated either through cell-cell contact or by a soluble mediator generated only during co-culture. To explore this, we repeated the oxidative stress assay, culturing BCCs alone or in the presence of medium pre-conditioned by a co-culture of BCCs and pre-Obs (CoCM). Interestingly, this CoCM still provided significant protection of BCCs from the cytotoxic effects of H_2_O_2_ (Figure 4d), confirming that the protective factor was a paracrine mediator produced as a result of cross-talk between the two cell types. As the protective effect was maintained following heat inactivation of the CoCM, (CoCM^HI^) (Figure 4e), we concluded that the paracrine protective factor was a low molecular weight, non-protein, mediator. One potential candidate molecule is glutathione, a small tripeptide that functions as an anti-oxidant in many organisms [33]. We therefore generated glutathione-depleted CoCM by addition of buthioinine sulfoximine (BSO), a non-reversible inhibitor of γ-glutamylcysteine synthase (the rate-limiting enzyme of the glutathione biosynthetic pathway) [34]. The glutathione-depleted CoCM still provided significant BCC protection against oxidative stress (Figure 4f), excluding glutathione as a protective factor. Although further work is required to identify the low molecular weight anti-oxidant acting as the paracrine protective factor, the results demonstrate that cells of the osteoblast lineage have the capacity to support survival of breast cancer cells undergoing stress. Interestingly, the paracrine protective mechanism induced by cross-talk between osteoblasts and TCs appears to be restricted to the TNBC subtype, as CoCM generated from either MDA-MB-231 (TNBC) or MCF7 (ER^+^) cells, which can also form bone metastases in mice, did not protect MCF7 cells from the cytotoxic effects of H_2_O_2_ (Figure 4g–h).

### 2.5. Osteoblast-Derived Juxtacrine Signaling Protects Breast Cancer Cells from Cell Stress

**Figure 5:**
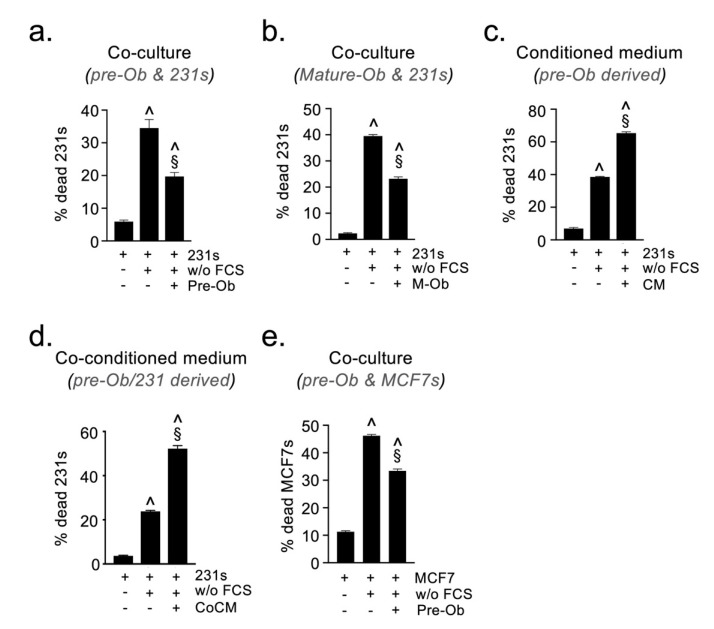
Osteoblast-derived juxtacrine signals protect hormone receptor positive and negative BCCs from serum-deprivation induced cell death. Flow cytometric quantification MDA-MB-231 viability in serum-starvation assays performed in the presence of (**a**) pre-osteoblastic cells, (**b**) mature osteoblasts (**c**) pre-Ob conditioned medium, (**d**) pre-Ob and MDA-MB-231 co-conditioned medium. (**e**) Quantification of MCF7 viability in serum-starvation assays performed in the presence of pre-Ob. (w/o FCS = without FCS, M-Ob = mature osteoblasts; ^ sig. dif. w.r.t. BCCs in full serum (*p* < 0.001), § sig. dif. w.r.t. BCC without serum (*p* < 0.001); *n* = 4 per condition).

The growth factor and cytokine milieu of the various metastatic organs is often markedly different from one another and the primary tumor, DTCs arriving at such sites may be exposed to stresses associated with growth factor deprivation. To mimic this process, we established a co-culture assay in which BCCs underwent serum-starvation for 48 h in the presence of pre-Obs or mature Obs, and their viability was quantified by flow cytometry as described in the Materials and Methods. Similar to oxidative stress, the presence of pre-Obs had a significant protective effect on BCCs exposed to serum-deprivation (Figure 5a). The same effect was observed when using mature osteoblasts (Figure 5b). The protective effect could not be recapitulated using either CM or CoCM (Figure 5c,d), suggesting that the protection is conveyed via a juxtacrine (contact-dependent) mechanism. This juxtacrine protective effect of pre-osteoblasts was also observed when using ER^+^ MCF7 cells, which suggested a common mechanism (Figure 5e). Integrins (α_v_ and β_1_) and notch-mediated juxtacrine signaling in bone is known to control the proliferation of DTCs [7,35,36,37]. Therefore, we investigated whether either of these pathways was responsible for the protective activity of pre-Obs under serum-starvation. Neutralizing antibodies against α_v_ and β_1_ integrins, or the γ-secretase inhibitor ‘DAPT’, did not reverse the protective effect, ruling out a role for these molecules in supporting DTC survival under stress (Figure S3).

**Figure 6:**
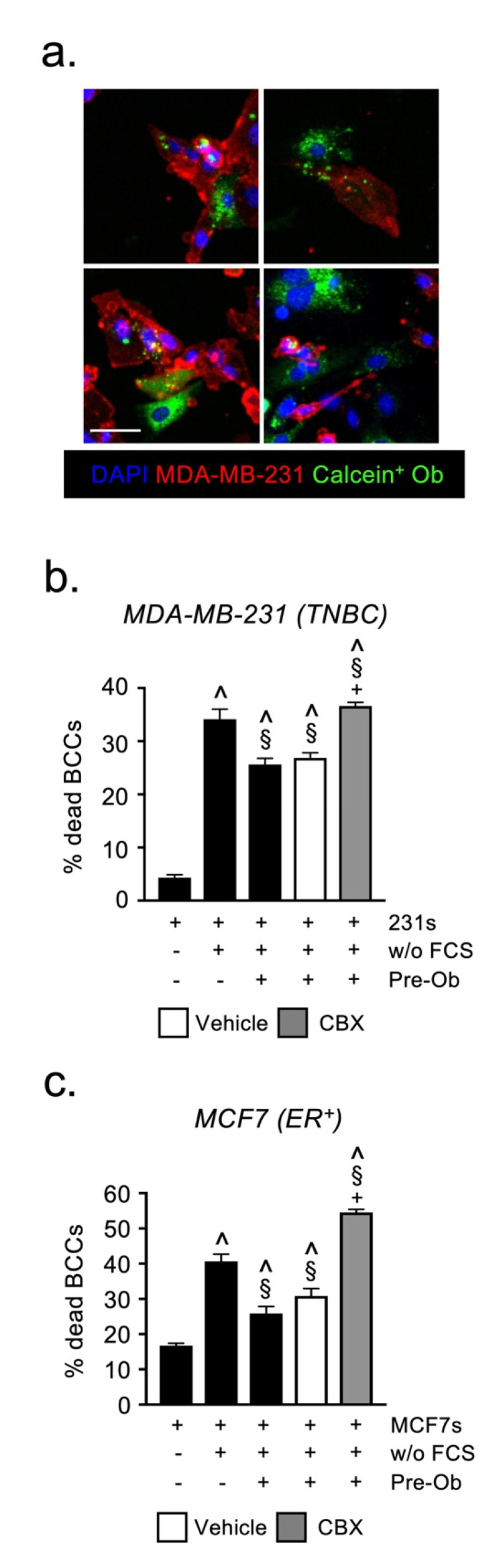
Gap junction signaling with osteoblasts protects BCCs from serum deprivation-induced cell death. (**a**) Representative images of gap junctional-mediated calcein transfer from labeled pre-Ob to MDA-MB-231 cells, in vitro. Flow cytometric quantification of (**b**) MDA-MB-231 and (**c**) MCF7 under serum-free conditions in the presence of pre-Ob and the gap junction inhibitor carbenoxolone or vehicle. (CBX = Carbenoxolone, w/o FCS = without FCS; scale bar = 50 µm; ^ sig. dif. w.r.t. BCCs under full serum (*p* < 0.0001), § sig. dif. w.r.t. BCCs under serum-free conditions (*p* < 0.001), + sig. dif. w.r.t. vehicle (*p* < 0.001); *n* = 4).

We hypothesized that the formation of gap junction complexes allows pre-Obs and BCCs to share protective mediators, thereby shielding both cell-types from the cytotoxic effects of serum-starvation. To confirm that gap junctions were forming between pre-Obs and BCCs, we loaded pre-Obs with calcein-AM and co-cultured them with MDA-MB-231 cells for 24 h. Using confocal microscopy, we observed evidence of calcein transfer from pre-Obs to MDA-MB-231, thereby demonstrating the formation of functional gap junction complexes between these two cell types (Figure 6a).

We then repeated the serum-starvation assays in the presence of the gap junction inhibitor Carbenoxolone (CBX). Preventing gap junction formation with CBX significantly reversed the protective effect exerted by pre-Obs on both TNBC and ER^pos^ BCCs exposed to serum starvation (Figure 6b,c). These experiments are direct evidence that the exchange of material through gap junctions is essential for the survival of cancer cells exposed to cell stress, supporting a role for pre-OBs in the bone metastatic niche.

### 2.6. Candidate Growth Factors, Cytokines or Angiogenic Mediator Expression Does Not Explain Differences in the Fate of DTCs

In order to identify potential mechanisms involved in regulating DTC indolence/outgrowth, we carried out a transcriptomic and proteomic comparison of the bone microenvironment in the two models. Quantitative real-time PCR was used to measure the expression of 48 candidate metastasis and bone biology-related genes (Table S1) and levels of 108 different growth factors, cytokines and angiogenic mediators compared by protein array. In the indolence model we found biologically relevant increases in the expression of five genes compared with the outgrowth model, *Spp1* (*Osteopontin*), *Tnc* (*Tenascin-C*), *Postn* (*Periostin*) and *Tnfsf11* (*Rankl*) and *Tnfrsf11b* (*Osteoprotegerin*) (Figure S1A). Of these, *Spp1* and *Tnfrsf11b* have been linked to metastatic dormancy [38,39] whereas *Tnc*, *Postn* and *Tnfsf11,* paradoxically, have been implicated as drivers of outgrowth, not dormancy/indolence [9,17].

The protein array revealed a semi-quantitative increase in the expression of five proteins; Cxcl4, FasL, Cxcl11, Cxcl15 and Tnfrsf11b, in the indolence compared with the outgrowth model (Figure S1B). As there is no Cxcl15 orthologue in humans, this molecule was not pursued. To further explore whether these mediators may have a role in determining the fate of DTCs, we examined the expression of their receptors on MDA-MB-231 cells, in vitro. Despite absent/low cell surface expression, all receptors could be detected as an intra-cellular pool, suggesting that MDA-MB-231 cells have the ability to respond to these cytokines following receptor shuttling to the cell surface (data not shown). We further validated the results of the protein array by quantifying Cxcl4, Cxcl11, FasL and Tnfrsf11b expression by ELISA, in bone marrow protein lysates prepared from the indolence and outgrowth models. In the indolence model there was a statistically significant increase in the expression of Cxcl4, Tnfsf11b, but not Cxcl11 or FasL, compared to the outgrowth model (Figure S1C). The high level of basal Cxcl4 expression in the outgrowth model, combined with the incremental increase found in the indolence model, suggests that this cytokine is unlikely to act as the molecular switch controlling metastatic latency in bone. However, the elevated expression of Tnfrsf11b in the indolence model implicates suppressed bone turnover as an important negative regulator of metastatic outgrowth in our models.

## 3. Discussion

The current study presents novel data demonstrating that following initial bone colonization, DTCs survive in micro-anatomical niches that contain an abundance of osteoblastic cells. We also show that the ability of the bone microenvironment to support large numbers of DTCs declines when the number of osteoblastic cells is reduced during skeletal maturation. Furthermore, we show that both paracrine and juxtacrine signaling with osteoblasts protects breast cancer cells from stress-induced death. Of particular interest, heterotypic gap junction signaling with osteoblasts was found to protect both TNBC and ER^pos^ subtypes of breast cancer from stress induced by serum deprivation and may, therefore, represent a common mechanism promoting the survival of breast cancer cells in bone.

Our analysis revealed a decline in osteoblastic cells in the metaphyseal compartment of long bones in the indolence model (mature mice, low bone turnover) compared with the outgrowth model (young mice, high bone turnover). These observations were consistent with previously published studies describing the age-related decline in osteoblastic cells in mice [10,26]. If these cells provide important proliferative cues to DTCs in bone, their decline in mature mice could explain the absence of DTC outgrowth in this model. Interestingly, we found that DTCs in both outgrowth and indolence models occupied niches that contained equivalent numbers of osteoblastic cells, demonstrating that DTCs survived predominantly in osteoblast-rich bone niches. The adult human skeleton contains low numbers of osteoblastic cells that are distributed throughout the skeleton at sites of active bone turnover, which occurs at a much slower rate than during adolescence [40,41]. If breast cancer cells arriving in human bone need osteoblastic cells to survive, this might partly explain the inefficiency of the metastatic process and the diffuse multi-focal pattern of skeletal metastases observed in patients. The abundance of bone resorbing osteoclasts also declines with age in our mouse models, we and others have already established the important role these cells play in supporting metastatic outgrowth in bone [17,28], hence osteoclasts were not the focus of the current study.

Taken together, these data suggested that the interaction with osteoblastic cells in bone may provide important survival cues for MDA-MB-231 cells (TNBC), though may not influence their subsequent fate, i.e., to proliferate or not. The observation that osteoblastic cells constitute part of the metastatic niche in bone has been reported previously [5,13,36,42,43,44,45]. The formation of heterotypic adherens junctions with N-cadherin^pos^ osteoblasts provides an important proliferative cue to E-cadherin^pos^ ER^pos^ breast cancer cells (MCF7), promoting their growth to form overt metastases in bone [5]. However, the role of such interactions in promoting breast cancer cell survival and resistance to stress has not previously been investigated, nor is this mitogenic interaction applicable to all breast cancer subtypes e.g., TNBC [5].

Gap junctions are intercellular membrane channels that permit the exchange of small molecules, ions and metabolites between adjacent cells. They are usually formed between cells of the same type (homotypic) via the interaction of two hexameric hemi-channels called connexons (one hemi-channel on each cell), which in humans can be constructed from any one of 20 connexin proteins. There is also evidence that heterotypic gap junctions can form between breast cancer cells and osteoblasts [36,46]. Recent elegant studies by Wang and colleagues (2018) demonstrated that the formation of heterotypic gap junctions with osteoblasts conferred a proliferative advantage on ER^pos^ breast cancer cells in bone, driving tumor growth and the formation of overt metastases [13]. Similar proliferative interactions exist between breast and lung cancer cells that metastasize to the brain, where they form heterotypic gap junctions with astrocytes [47]. However, the role of such interactions in supporting the survival of different subtypes of breast cancer cell in bone in the early stages following dissemination, rather than stimulating proliferation and growth to form tumors, was not addressed. In the present study, we have expanded on these important findings by demonstrating that the formation of gap junctions between osteoblasts and either MDA-MB-231 (TNBC) or MCF7 (ER^pos^) breast cancer cells conveyed significant protection against stress-induced cell death triggers of the types encountered along the metastatic cascade. To our knowledge, this is the first demonstration that heterotypic gap junction signaling may function at an early stage after the initial arrival of cancer cells in bone, by providing DTCs with an enhanced ability to survive the stresses associated with the metastatic process.

We also demonstrated that paracrine signaling can protect breast cancer cells from stress-induced cell death. Our data revealed that cross-talk between osteoblastic cells and MDA-MB-231 (TNBC) cells resulted in the production of an as yet unidentified soluble, thermally-resistant mediator(s), possibly an active metabolite, that conveyed significant protection against oxidative stress. These paracrine factors were not released by osteoblasts cultured in isolation and were not induced by co-culture with MCF7 (ER^pos^) breast cancer cells. These data indicate that whilst there are common mechanisms by which these two cell lines, representing different subtypes of breast cancer, resist stress-induced cell death (i.e., through formation of heterotypic gap junctions), there are other as yet unidentified processes that might be specific to both the breast cancer subtype and the type of stress encountered. Our observation that the protective mechanisms required either direct physical contact between breast cancer cells and osteoblastic cells or an ability to communicate with one another, highlights the importance of using a co-culture approach, rather than monoculture-conditioned medium assays, for characterizing such protective processes, in vitro.

Our data demonstrate that stress-specific mechanisms of resistance to cell death are deployed by BCCs and osteoblasts, however not every resistance mechanism is possessed by each BCC subtype. MDA-MB-231 cells (TNBC) were able to elicit the support of osteoblasts and thereby resist cell death induced by both oxidative stress (paracrine mechanism) and growth factor deprivation (juxtacrine mechanism). In a translational setting, we would speculate that possessing multiple mechanisms of stress resistance would make TNBC bone metastases more difficult to treat than ER^pos^ metastases. We found that gap junctional signaling between MDA-MB-231 tumor cells and osteoblasts was important in protecting against growth factor deprivation. However, it is not clear if targeting this support mechanism alone would have efficacy in cancer patients. Additional work is required to identify the paracrine mechanisms mediating resistance to oxidative stress in TNBC (i.e., revealing the identity of either the BCC-secreted factor responsible for educating osteoblasts or the protective factor itself). The most effective treatment strategy for TNBC-derived bone metastasis is likely to require simultaneous targeting of resistance mechanisms against multiple forms of cell stress. Interestingly, MCF7 cells (ER^pos^) showed an enhanced level of cell death induced by oxidative stress. This finding is in agreement with reports that MCF7 cells (ER^pos^) are sensitive to oxidative stress which arises from their low endogenous levels of some antioxidants [48], which is further compounded by estrogen signaling [49]. In addition, like the majority of breast cancers, MCF7 cells express both ER and wildtype, functional p53, which renders MCF7 cells sensitive to cell death induced by oxidative stress [49,50,51]. However, it remains to be determined whether p53 status directly influences the ability of breast cancer cells to engage in paracrine crosstalk with osteoblasts. Nevertheless, our data demonstrate that the sensitivity of MCF7 cells to oxidative stress in co-culture, and possibly in vivo, arises from their inability to secrete the paracrine mediators necessary for eliciting the support of osteoblasts. Taken together, these data highlight a potential therapeutic vulnerability in ER^pos^ bone metastasis that might be exploitable in a clinical setting.

We observed that between 70–80% of DTCs in bone were associated with the microvasculature, regardless of their subsequent fate. These data are consistent with the established importance of the perivascular niche in bone metastasis [3,7,8,9]. However, our data also showed that a significant number of these perivascular DTCs were simultaneously associated with the endosteal niche. This finding highlights the importance of overlapping niches and the complex interplay between their various cellular components during breast cancer metastasis to bone. This concept is supported by the observation that the successful recreation of perivascular niches capable of supporting breast cancer cell dormancy in vitro requires that endothelial cells be co-cultured with bone-derived osteoblast precursors [7,9]. A greater understanding of the cross-talk between this cell triad may shed further light on the mechanisms promoting the survival of breast cancer cells in bone and reveal additional avenues for therapeutic exploitation.

Regarding the bone microvasculature, type-H^CD31pos^ vessels comprise proliferative endothelial cells that regulate osteoblastogenesis and thereby couple the processes of angiogenesis and osteogenesis in vivo [10]. Consequently, type-H vessels are more abundant in young mice with ongoing skeletal development, but decline with age [10,26]. Therefore, we speculated whether this type of vessel is associated with metastatic outgrowth. However, DTCs associated with either type-H or -L vessels exhibited metastatic outgrowth, and metastatic latency occurred even when up to 50% of DTCs were associated with type-H vessels in mature mice. Taken together, these data demonstrate that the fate of DTCs in bone, i.e., to grow or not, is not determined by their association with a specific subtype of blood vessel. However, given their role in osteoblastogenesis, we cannot exclude the possibility that type-H vessels may function as indirect, upstream regulators of breast cancer cell survival in bone via their effects on osteoblastic cells.

Finally, our transcriptomic and proteomic profiling of candidate molecules did not reveal any differentially expressed, biologically relevant, factors that could directly influence the fate of DTCs, between the bone microenvironments of the outgrowth and indolence models. However, there was increased osteoprotegerin expression at both mRNA and protein level in the bone microenvironment of the indolence model. Osteoprotegerin is a decoy receptor that sequesters and inactivates RANKL, thereby preventing osteoclast-driven bone resorption, suggesting that a decline in bone turnover in mature mice may be a cause of metastatic latency. This is consistent with our work and that of others showing that increased osteoclast-driven bone resorption can trigger the growth of previously indolent bone metastases, in pre-clinical models [17,28,39]. However, in our models we did not detect differential expression of growth factors commonly sequestered in bone (e.g., Igf, Pdgf, Fgf or Tgfb1), which may drive metastatic outgrowth if released during bone turnover. These data suggest that the ability to grow and form overt bone metastases might be an intrinsic property that is restricted to specific DTC clones, rather than being determined directly and solely by the niche, which is supported by our mathematical modelling. Indeed, the modelling suggests that the endogenous factors (i.e., metabolites or immune-mediated phenomenon) within the bone niche may only make a minor contribution to the outgrowth of DTCs. We postulate that the decline in osteoblastic cells in the indolence model reduces the total number of DTCs that can survive in bone, thus reducing the likelihood of an outgrowth competent clone finding a supportive niche. These data are entirely consistent with work from the Massague [27] and Kang [28] labs, demonstrating that only specific cancer cell clones possess the ability to drive osteoclast-mediated bone resorption and thereby fuel metastatic outgrowth. Taken together, our study identifies a novel process by which osteoblasts might act as upstream regulators of metastatic latency in bone. By protecting DTCs against the mediators of metastasis-associated cell stress, osteoblasts determine the number of supportive niches in bone available for colonization by cancer cell clones competent of metastatic outgrowth. While additional work is required to identify the paracrine mediator(s) that specifically protect MDA-MB-231 (TNBC) cells from oxidative stress, gap junctional (juxtacrine) signaling represents a protective mechanism deployed by both TNBC and ER^pos^ subtypes of breast cancer. Further comprehensive in vitro and in vivo studies are required to identify the protective pro-survival factors transferred by gap junctions and the precise connexin proteins involved. Our novel data suggest that the potential of combining gap junction inhibitors with current standard of care should be explored in preclinical studies as a novel therapeutic approach for the adjuvant treatment of breast cancer.

## 4. Materials and Methods

### 4.1. Animal Experiments

All animal experiments were conducted with the approval of the University of Sheffield Project Applications and Amendments (Ethics) Committee and in accordance with UK Home Office Regulations (PPL70/8964 to N.J.B.). BALB/c Nude [Foxn1-Crl] immunodeficient mice (Charles River Laboratories, Wilmington, MA, USA) were housed in individual ventilated cages with 12-h light/dark cycles at 22 °C and access to food and water ad libitum. Experimental bone metastases were established by intra-cardiac injection of 1 × 10^5^ MDA-MB-231 cells, as described previously [17,18]. Mice were culled six days post-injection for analysis before metastatic outgrowth occurs [18]. Ovariectomy was performed as described previously [17]. One week after removal of the ovaries, or sham operation, experimental bone metastases were established, and the mice were culled six days post-injection.

### 4.2. Cell Lines and Tissue Culture, Reagents

Breast cancer cell lines MDA-MB-231^GFP^ and MCF7 (ATCC) were cultured in RPMI-1640 medium (Thermo Fisher Scientific, Waltham, MA, USA) containing 10% FCS. Mouse pre-osteoblastic cell line MC3T3-E1 (ECACC) was cultured in αMEM medium (Thermo Fisher Scientific) containing ribonucleosides, deoxyribonucleosides and 10% FCS. To generate mature osteoblasts, MC3T3-E1 cells were differentiated for 3 weeks in αMEM containing 2mM β-glycerophosphate, 50 µg/mL ascorbate and 10% FCS. All cell lines were passaged at 80% confluence. Details of the reagents used are included in Supplementary Table S2.

### 4.3. Immunofluorescent Analysis

Gelatin-embedded hind limb cryo-sections were labeled as described previously [26]. Briefly, cryo-sections were blocked with a streptavidin/biotin blocking kit (Vector Laboratories, Birlingame, CA, USA), incubated for 1.5–2 h with directly conjugated or unconjugated primary antibodies (2 µg/mL) followed by 1 h with either biotinylated (7.5 µg/mL) or fluorescent (5 µg/mL) secondary antibodies. Where appropriate, slides were incubated for 20 min with fluorescently conjugated streptavidin (10 µg/mL). Labelled sections were stained with DAPI and mounted in ProLong Diamond Antifade (Thermo Fisher Scientific, Waltham, MA, USA). Primary antibodies were directed against mouse Endomucin (Santa Cruz Biotechnology, Dallas, TX, USA), CD31 (Dianova, New York, NY, USA), Osteopontin (RandD Systems, Minneapolis, MN, USA), Osterix (Abcam, Cambridge, MA, USA) and human CD29 and CD59 (BioLegend, San Diego, CA, USA). Image were acquired using a Nikon A1 confocal microscopy (Nikon, Tokyo, Japan) and the images were analyzed using ImageJ (National Institutes of Health [NIH], Bethesda, MD, USA). To determine the abundance of osteoprogenitors in proximity to DTCs (‘Hotspot analysis’) high powered fields (HPF) of view were acquired using a 40× oil immersion objective lens. Each HPF was centered on the DTCs and the numbers of osteoprogenitors present was quantified.

### 4.4. Oxidative Stress and Serum Deprivation Assays

For both assays, MC3T3-E1 cells were grown to confluence in 12-well plates. For oxidative stress assays, BCCs (5 × 10^4^ cells/well) were seed onto confluent MC3T3-E1s and 24 h later co-cultures were treated with 400 µM H_2_O_2_. BCC viability was determined 24 h post-treatment by flow cytometry by addition of the cell impermeant DNA-binding dye TO-PRO-3^TM^, which labels the nuclei of dead cells [52,53]. For serum-starvation assays, BCCs were seeded directly onto confluent MC3T3-E1s in serum-free αMEM at a density of 1 × 10^5^/well and incubated for 48 h and tumor cell viability was determined by flow cytometry. To generate conditioned medium, confluent monolayers of MC3T3-E1 cells were cultured alone and in combination with 1 × 10^5^ BCCs in either full serum or serum-free αMEM for 48 h. GSH was depleted from co-culture conditioned medium using the γ-Glutamylcysteine synthase inhibitor ”Buthionine Sulfoximine” (BSO—100µM). Co-cultures were treated with BSO for 24 h, the medium was then replaced, and conditioned medium collected overnight in the absence of BSO. Gap junction, notch and integrin-mediated signaling pathways were inhibited in co-cultures using Carbenoxolone (25–50 µM), the γ-secretase inhibitor ”DAPT” (1 µM) and integrin-neutralizing antibodies (10–50 µg/mL), respectively. Inhibitors were added to co-culture assays at the time of tumor cell seeding. Each test condition was measured in triplicate in three independent experiments.

### 4.5. Protein Array and ELISA

Protein lysates were generated from snap-frozen femurs as described previously [26]. Mouse ”Growth Factor Array C3“, ”Angiogenesis Array C1“ and ”Cytokine Array C1000“ (RayBiotech, Peachtree Corners, GA, USA) were incubated with 1 mg of total protein pooled from four mice (250 µg protein/mouse) aged 6-week and 12-week. Membranes were developed using a chemiluminescent substrate and GelDoc imaging system (BioRad Laboratories, Hercules, CA, USA). Densitometric analysis or protein expression was performed using ImageJ software (National Institutes of Health [NIH], Bethesda, MD, USA). Changes in expression ~50% were deemed biologically relevant. Colorimetric ELISAs for mouse Cxcl4 (Abcam, Cambridge, MA, USA), Cxcl11 and Cxcl15 (RayBiotech, Peachtree Corners, GA, USA), Collagen IV (Bio-techne, Minneapolis, MN, USA), and FasL (Cambridge Bioscience, Cambridge, UK) were conducted using protein lysates from 4 individual mice aged 6-week and 12-week-old, samples from each mouse were measured in triplicate, after first adjusting the total protein concentration of each sample to 4 mg/mL by dilution with PBS.

### 4.6. Gap Junction-Mediated Transfer of Calcein

MC3T3-E1 cells were suspended in PBS containing calcein-AM (1 µM) for 20 min at 37 °C, washed thoroughly with PBS and seeded at a 1:1 ratio with MDA-MB-231 (5 × 10^4^ cells/well) in αMEM for 24 h. Calcein transfer was imaged using a Nikon A1 confocal microscope.

### 4.7. Real-Time Quantitative PCR

RNA was extracted from marrow and bone-lining cells from mouse tibias as described previously [26] and 1 µg of total RNA reverse transcribed using a QuantiTect Reverse Transcription Kit (Qiagen, Hilden, Germany). Gene expression was determined using in-house validated SYBR green primers (sequences specified in Supplementary Table S1) and an HT7900 real-time PCR instrument (Thermo Fisher Scientific). Changes in gene expression were calculated using a relative quantification approach (∆∆Ct) after first normalizing to the housekeeping gene glyceraldehyde 3-phosphate dehydrogenase (GAPDH) (Table S1). Experiments were performed using four mice in each treatment group. The expression of each gene of interest was measured in triplicate for each mouse (*n* = 4).

### 4.8. Mathematical Modelling of Outgrowth Probability

The birth and death of cancer cells was modelled as a continuous time Markov chain using a discrete random variable *N(t)* number of DTCs at time *t* and constant birth and death rate parameters such that the birth and death rates are linearly proportional to the population number *N.* We solved the differential equation using a probability generation function to find the extinction probability at long times, *P(tumor) = 1 − A^n^,* where *A* is the ratio of death rate to proliferation rate and *n* is the initial number of DTCs (see Figure S2 for further details).

### 4.9. Statistical Analysis

Statistical analyses were performed using Prism software (v.8.0; GraphPad Software, Irvine, CA, USA). All data are presented as sample means ± SEM and all data were analyzed by one- or two-tailed student’s *t*-test, or one-way ANOVA, where appropriate. A statistically significant difference was defined as *p* < 0.05.

## 5. Conclusions

Our study deployed a range of molecular, cellular and in vivo methodologies to generate compelling evidence that osteoblasts act as regulators of metastatic outgrowth in bone by dictating the overall abundance of DTCs. Osteoblasts were found to deploy a range of paracrine and juxtacrine mechanism that protect DTCs from death induced by mediators of metastasis-induced cell stress, with gap junctional signaling being of particular interest. In addition to the established role for heterotypic gap junction signaling in promoting the proliferation of ER^pos^ DTCs in pre-clinical mouse models, we now extend this work by demonstrating their role as conveyors of osteoblast-derived cues that promote breast cancer cell resistance to cell stress. Taken together, our data indicate that targeting the interaction between DTCs and osteoblasts, particularly gap junctional signaling, could be explored as a therapeutic approach to eliminate minimal residual disease.

## Data Availability

Data are contained within this article or Supplementary Materials.

## Supplementary Materials

The following are available online at https://www.mdpi.com/2072-6694/13/6/1366/s1, Figure S1: Transcriptomic and proteomic profiling on bone marrow from outgrowth and indolence mouse models, Figure S2: Modelling Tumor Growth. Figure S3: Targeting ITAV, ITGB1 and γ-secretase does not reverse the ability of osteoblasts to protect tumor cells from serum deprivation-induced cell death. Table S1: Gene expression profiling data. Table S2: Reagents list and supplier details.
