# Supplementary material for: Osteoblast-Derived Paracrine and Juxtacrine Signals Protect Disseminated Breast Cancer Cells from Stress
Source: Cancers (Basel). 2021 Mar 18;13(6):1366. doi: 10.3390/cancers13061366 (PMC8003019; doi:10.3390/cancers13061366)
Supplement: Supplementary file 1 [file cancers-13-01366-s001.zip › Figure S1.docx]

**Figure S1.**

b.

**Cxcl4**

**Fasl**

**Cxcl11**

**Cxcl15**

0

2.5

5.0

10.0

12.5

15.0

17.5

20.0

Arbitrary units (x10^3^)

Outgrowth (6-week)

Indolence (12-week)

c.

***Figure S1. Transcriptomic and proteomic profiling on bone marrow from outgrowth and indolence mouse models.*** (**a**) Transcriptomic profile of 48 genes expressed as Log_2_ fold change in indolence compared to outgrowth mouse models. Red line denotes upper threshold for upregulation, blue line denotes lower threshold for downregulation. (**b**) Semi-quantitative, densitometric proteomic profiling of pooled bone marrow lysates from indolence and outgrowth mouse models (*left*) alongside representative protein arrays (*right*). (**c**) ELISA-based quantification of Cxcl4, Cxcl11 and Fasl protein lysates from individual indolence and outgrowth mice. (*OutG. = outgrowth, Indol. = indolence; ^ sig. dif. w.r.t. outgrowth (P<0.05); n = 4 mice per condition*).

a.

6

Log_2_ FC (w.r.t Outgrowth)

0

2

4

-2

-4

-6

*Spp1*

*Tnc*

*Postn*

*Tnfsf11*

*Tnfrsf11b*

**Tnfrsf11b**

Cytokine Array


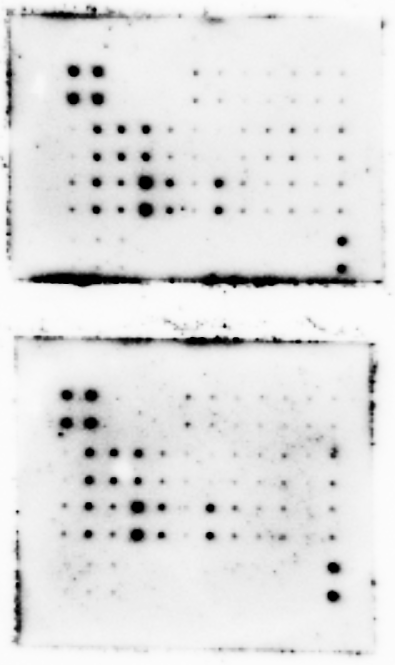

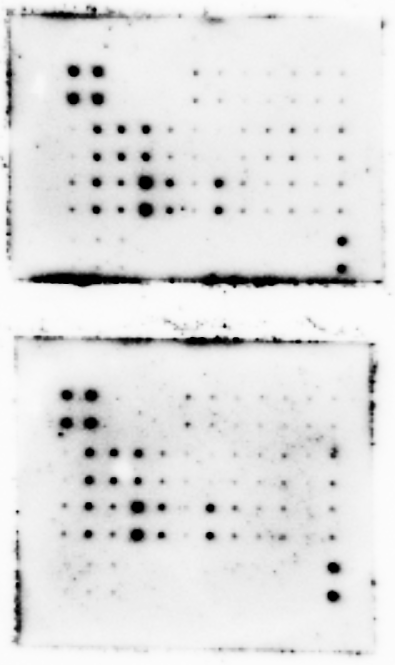

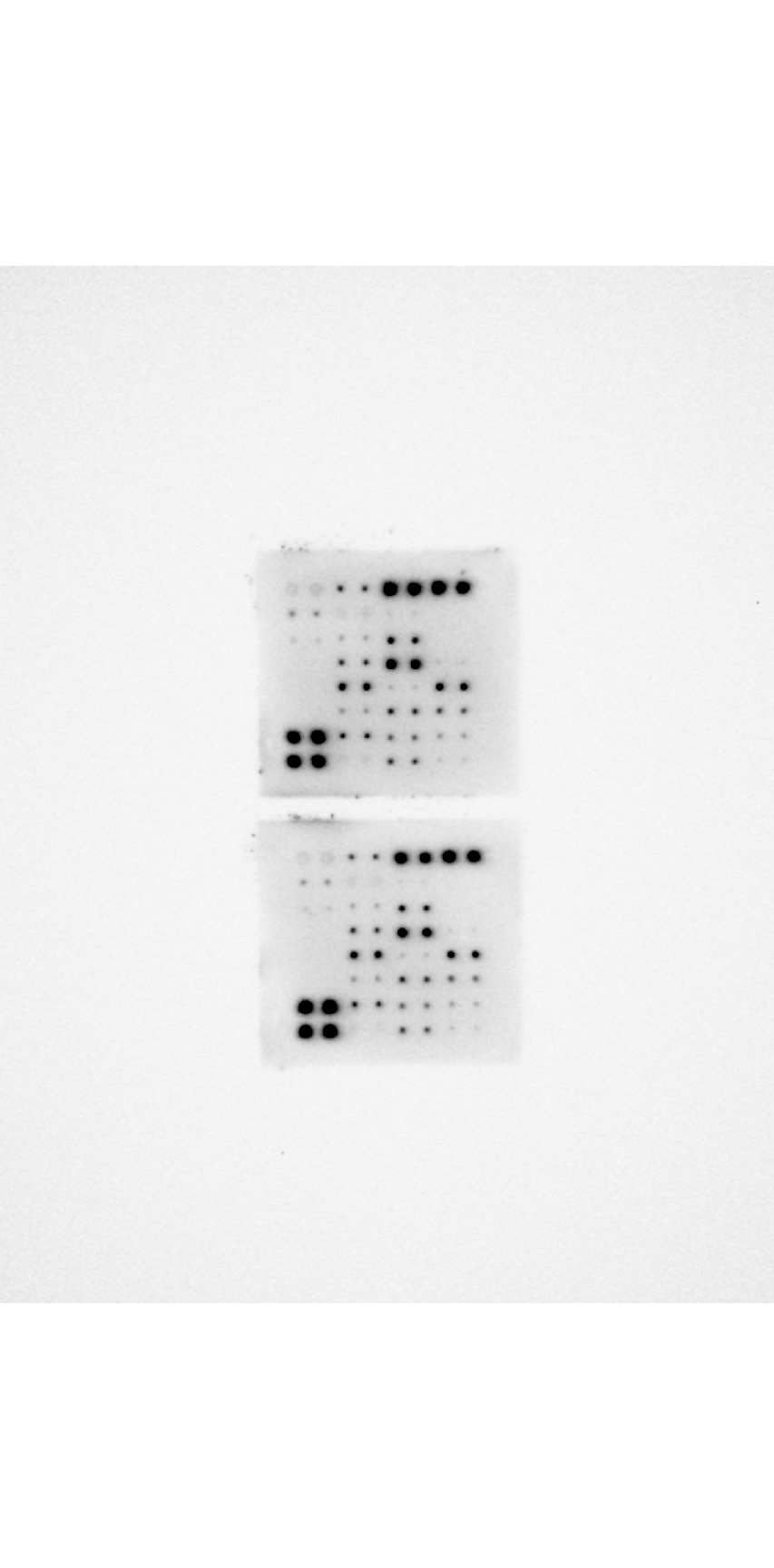

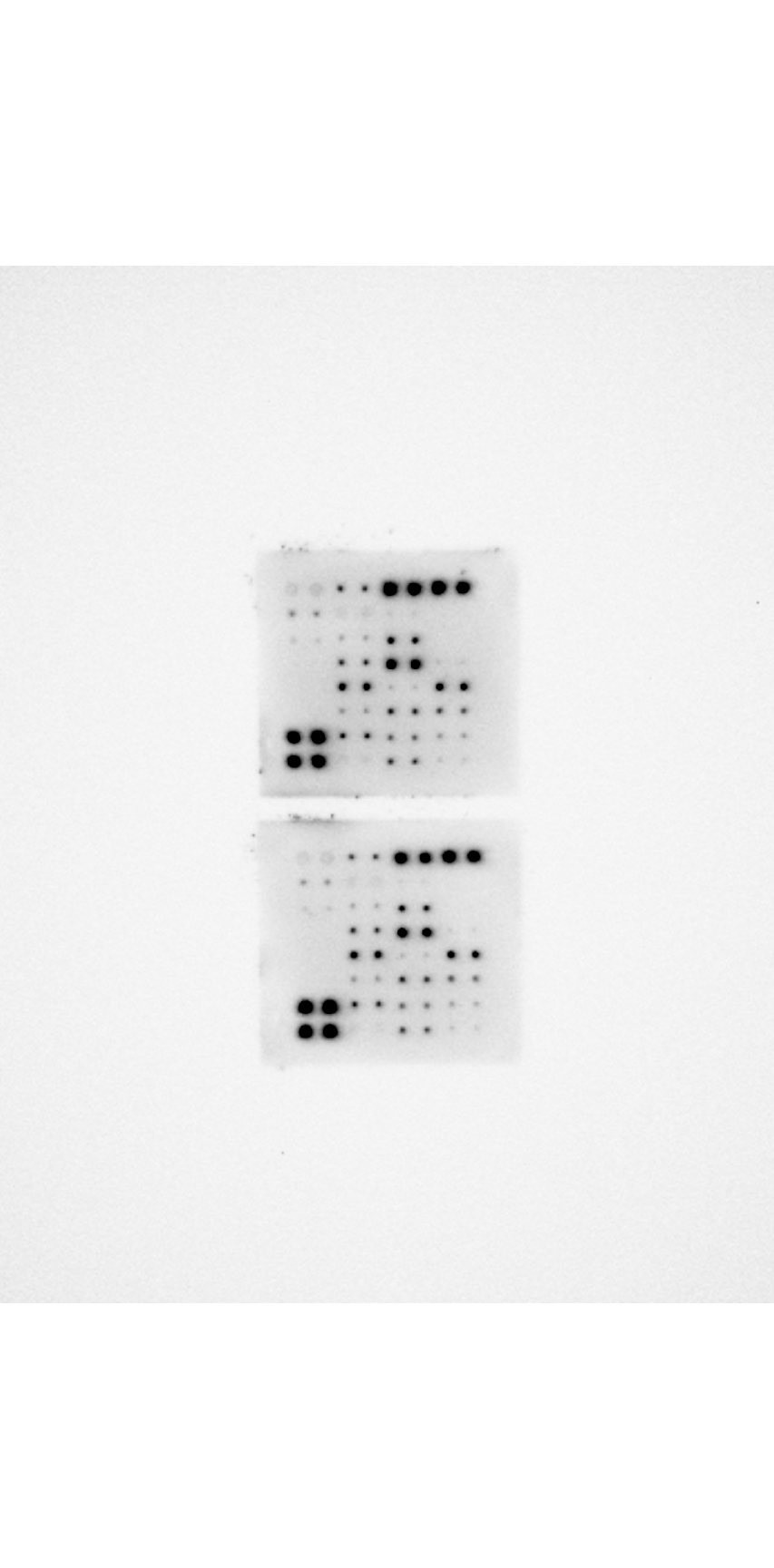


Angiogenesis Array

*Outgrowth*

*Indolence*

Cxcl4

0

2

4

6

(ng/mL)

OutG.

Indol.

^

Cxcl11

0

2

4

6

8

10

(pg/mL)

OutG.

Indol.

Fasl

0

5

10

15

(pg/mL)

OutG.

Indol.

OutG.

Indol.

Tnfrsf11b

1000

750

500

250

0

(pg/mL)

^
