# Supplementary material for: Osteoblast-Derived Paracrine and Juxtacrine Signals Protect Disseminated Breast Cancer Cells from Stress
Source: Cancers (Basel). 2021 Mar 18;13(6):1366. doi: 10.3390/cancers13061366 (PMC8003019; doi:10.3390/cancers13061366)
Supplement: Supplementary file 1 [file cancers-13-01366-s001.zip › Figure S3.docx]

**Figure S3**

ITGAV targeting

0

10

20

30

40

% dead 231ss

^

§

^

§

^

§

^

+

+

+

+

+

+

+

+

+

+

+

+

-

-

-

231s

Pre-Ob

IgG_2a_ control

Anti-ITGAV

a.

b.

0

10

20

30

40

50

% dead 231s

+

+

+

+

+

+

+

+

+

+

+

+

-

-

-

231s

Pre-Ob

w/o FCS

IgG_1_ control

Anti-ITGB1

^

§

^

§

^

§

^

ITGB1 targeting

w/o FCS

c.

0

10

20

30

40

50

60

% dead 231s

+

+

+

+

+

+

+

+

+

+

+

+

-

-

-

231s

Pre-Ob

w/o FCS

+

+

+

+

+

+

Vehicle

1 µM DAPT

0.1 µM DAPT

*γ-*secretase inhibition

^

§

^

§

^

^

^

^

***Figure S3. Targeting ITGAV, ITGB1 and γ-secretase does not reverse the ability of osteoblasts to protect tumour cells from serum deprivation-induced cell death.*** Flow cytometric quantification of MDA-MB-231 viability in serum-starvation assays performed in the presence of (**a**) neutralizing antibody again ITGAV, (**b**) neutralizing antibody against ITGB1 or (**c**) a *γ*-secretase inhibitor - DAPT. (*w/o FCS = without serum, Pre-Ob = pre-osteoblast; ^ sig. dif. w.r.t. BCCs in full serum (P<0.0001), § = sig. dif. w.r.t. BCCs without serum (P<0.01); n = 4 per condition*).
