# Supplementary material for: Osteoblast-Derived Paracrine and Juxtacrine Signals Protect Disseminated Breast Cancer Cells from Stress
Source: Cancers (Basel). 2021 Mar 18;13(6):1366. doi: 10.3390/cancers13061366 (PMC8003019; doi:10.3390/cancers13061366)
Supplement: Supplementary file 1 [file cancers-13-01366-s001.zip › Table S1.docx]

**Table S1**

Table S1. Gene expression profiling data.

| **Gene name** | **Log_2_ FC** | **SEM** | ***Fwd* Primer** | ***Rev* Primer** |
| --- | --- | --- | --- | --- |
| *Bmp7* | -1.41 | 0.22 | *TTTGACATCACAGCCACCAGCAAC* | *ATGAACCTCCGTGGCCTTGAAGAA* |
| *Sost* | -1.26 | 0.34 | *ACAACCAGACCATGAACCG* | *CAGGAAGCGGGTGTAGTG* |
| *Mmp2* | -1.25 | 0.53 | *ACAAGTGGTCCGCGTAAAGT* | *GTAAACAAGGCTTCATGGGGG* |
| *Dll4* | -1.02 | 0.26 | *GAACAGAGGTCCAAGCCGAA* | *CAGGCCCATTCTCCAGATCG* |
| *Hspg2* | -0.98 | 0.75 | *CAGACTCTGCGGATGGGATG* | *GGCTGTCTGTTGGCTAGGTT* |
| *Gas6* | -0.82 | 0.68 | *GGCTCAACTACACCCGAACA* | *TCGACTAGGGCCACAGAGAT* |
| *Scf* | -0.73 | 0.55 | *GAAGACACAAACTTGGATTATCACT* | *CCCGCAGATCTCCTTGGTTT* |
| *Ccl3* | -0.63 | 0.27 | *CCCTCTGTCACCTGCTCAAC* | *ATGGCGCTGAGAAGACTTGG* |
| *Bmp4* | -0.56 | 0.31 | *GCAACCCAGCCTGAGTATCT* | *ATGGCACTACGGAATGGCTC* |
| *Mmp9* | -0.56 | 0.15 | *CCGACTTTTGTGGTCTTCCCC* | *CAAAGCCGGCCGTAGAGAC* |
| *Nrp1* | -0.53 | 0.21 | *TTCAAGAGAGGGCCCGAATG* | *GTAGGTGCACTCCAAGCTGT* |
| *Fn1* | -0.53 | 0.61 | *ACGGTTTCCCATTACGCCAT* | *GGCACCATTTAGATGAATCGCA* |
| *Pthrp* | -0.50 | 0.34 | *CGCTGATTCCTACACAAGTCC* | *GACACTCCACTGCTGAACCA* |
| *Cdh2* | -0.43 | 0.18 | *TGTGGAGGCTTCTGGTGAAA* | *AATTTCACATTGAGAAGGGGCT* |
| *Col1a1* | -0.42 | 0.39 | *GGAAGAGCGGAGAGTACTGG* | *GACGGCTGAGTAGGGAACAC* |
| *Tgfb1* | -0.37 | 0.14 | *CGCAACAACGCCATCTATGAG* | *TTCCGTCTCCTTGGTTCAGC* |
| *Cxcl12* | -0.31 | 0.18 | *CCGCGCTCTGCATCAGT* | *GCGATGTGGCTCTCGAAGA* |
| *Fgf1* | -0.28 | 0.20 | *AAAGTGCGGGCGAAGTGTAT* | *CTCATTTGGTGTCTGCGAGC* |
| *Nes* | -0.26 | 0.49 | *GTCTACAGGCAGCGCTAACA* | *CAGCTCATGGGCATCTGTCA* |
| *Plgf* | -0.24 | 0.09 | *TTTATGTCAACGTTGCCCCC* | *GAGGCTGCAGTTCCCCATTA* |
| *Angpt1* | -0.20 | 0.23 | *AGCAGTACAACACCAACGCT* | *AATTCTCAAGTTTTTGCAGCCA* |
| *S100a8* | -0.20 | 0.10 | *CTTCAAGACATCGTTTGAAAGGAAA* | *TCTGCACAAACTGAGGACACT* |
| *Igf1* | -0.19 | 0.26 | *TAAGATCTGCCTCTGTGACTTCTT* | *CCTGTGGGCTTGTTGAAGTAAAA* |
| *Vcan* | -0.10 | 0.58 | *AAGGGAACAGTTGCTTGCGG* | *ATTGCCCATCTCCCGTTTCC* |
| *Pdgfa* | -0.09 | 0.26 | *GAGGAGGAGACAGATGTGAGG* | *AGGAGAACAAAGACCGCACG* |
| *Pdgfb* | -0.09 | 0.36 | *CAAGAGTGTGGGCAGGGTTAT* | *CCGAATCAGGCATCGAGACA* |
| *Csf1* | -0.04 | 0.35 | *CCCAACGAGTCAGCAACTCA* | *AATGCCCCAAGAGTGGCTTT* |
| *Tgfb2* | 0.00 | 0.24 | *ACCGCAACAACGCCATCTAT* | *ACCGCAACAACGCCATCTAT* |
| *Il1b* | 0.03 | 0.64 | *GCCACCTTTTGACAGTGATGAG* | *AGCTTCTCCACAGCCACAAT* |
| *S100a9* | 0.12 | 0.32 | *ACCACCATCATCGACACCTTC* | *AAAGGTTGCCAACTGTGCTTC* |
| *Angpt2* | 0.14 | 0.62 | *GCATGTGGTCCTTCCAACTT* | *GATCCTCAGCCACAACCTTC* |
| *Coco* | 0.20 | 0.17 | *TTTGTTCAGGTGATCTCCAGGC* | *AGCGGCCAAAACAGAGATGA* |
| *Axl* | 0.24 | 0.42 | *CCTTGAGCCAGTCCCCTATG* | *CACGATGGCCTGCAACTAAC* |
| *Tgfb3* | 0.25 | 0.35 | *GCACTTTACAACAGCACCCG* | *AGTTCATTGTGCTCCGCCA* |
| *Vegfa* | 0.32 | 0.23 | *CAGGCTGCTGTAACGATGAA* | *AATGCTTTCTCCGCTCTGAA* |
| *Il6* | 0.38 | 0.11 | *TCCAGTTGCCTTCTTGGGAC* | *TGCCATTGCACAACTCTTTTCTC* |
| *Cdh1* | 0.39 | 0.52 | *GGCTGGACCGAGAGAGTTAC* | *CCGGGCATTGACCTCATTCT* |
| *Cxcl12* | 0.57 | 0.52 | *TGCACCCAAACCGAAGTCAT* | *TGGGGACACCTTTTAGCATCT* |
| *Lox* | 0.60 | 0.69 | *TGGCCAGTTCAGCATATAGGG* | *TGGCTGAATTCGTCCATGCT* |
| *Vcam* | 0.67 | 0.20 | *TTTATGTCAACGTTGCCCCC* | *GAGGCTGCAGTTCCCCATTA* |
| *Tnfa* | 0.71 | 0.36 | *CCTTCACAGAGCAATGACTC* | *GTCTACTCCCAGGTTCTCTTC* |
| *Col4a1* | 0.88 | 0.56 | *CATTCAGATTCCGCAGTGCC* | *GGGCTTCTTGAACATCTCGCT* |
| *Il11* | 1.00 | 0.14 | *AACTGTGTTTGTCGCCTGGT* | *AAGCTGCAAAGATCCCAATG* |
| *Tnfrsf11b* | 3.06 | 1.34 | *GTGTGGAATAGATGTCACCCTGT* | *CTTGTGAGCTGTGTCTCCGT* |
| *Tnfsf11* | 4.06 | 0.52 | *CCCATCGGGTTCCCATAAAG* | *AGCAAATGTTGGCGTACAGG* |
| *Postn* | 4.14 | 0.84 | *GGAATTCGGCATTGTGGGAGCCACTACC* | *GGTCGACTCAAATTTGTGTCAGGACACGGTC* |
| *Tnc* | 4.40 | 0.43 | *ACCACAGAAGCTGAACCGGA* | *CAGTCCAGGACAGACGGAAA* |
| *Spp1* | 5.14 | 0.46 | *CTTTCACTCCAATCGTCCCTA* | *GCTCTCTTTGGAATGCTCAAG* |

Gene expression profiling data presented as Log_2_ fold change in the indolence model compared with the outgrowth model, including S.E.M. and SYBR green primer sequences
