# Supplementary material for: Osteoblast-Derived Paracrine and Juxtacrine Signals Protect Disseminated Breast Cancer Cells from Stress
Source: Cancers (Basel). 2021 Mar 18;13(6):1366. doi: 10.3390/cancers13061366 (PMC8003019; doi:10.3390/cancers13061366)
Supplement: Supplementary file 1 [file cancers-13-01366-s001.zip › Table S2.docx]

**Table S2**

**Table S2**. Reagents and supplier details

|  | **ITEM** | **SUPPLIER** | **CAT. NO:** |
| --- | --- | --- | --- |
| **ANTIBODIES** | Rat anti-mouse Endomucin | Santa Cruz Biotechnology | sc-65495 |
|  | PE-conjugated mouse anti-human CD29 | BioLegend | 303004 |
|  | PE-conjugated mouse anti-human CD59 | BioLegend | 304708 |
|  | Goat polyclonal anti-Osteopontin | R&D Systems | AF808 |
|  | Rat anti-mouse CD31 | Dianova | DIA-310 |
|  | Rabbit polyclonal anti-Osterix | Abcam | ab22552 |
|  | Biotin-conjugated goat anti-rabbit IgG | Thermo Fisher Scientific | SA5-10229 |
|  | Biotin-conjugated goat anti-rat IgG | Thermo Fisher Scientific | PA1-29608 |
|  | Alexa Fluor 647-conjugated Streptavidin | Thermo Fisher Scientific | S32357 |
|  | Alexa Fluor 488-conjugated goat anti-rat IgG | Thermo Fisher Scientific | A-11006 |
|  | Neutralising mouse anti-human ITGAV | BioLegend | 327911 |
|  | Neutralising mouse anti-human ITGB1 | R&D Systems | MAB17781 |
| **ARRAYS** | Mouse Growth Factor Array C3 | RayBiotech | AAM-GF-3-2 |
|  | Mouse Angiogenesis Array C1 | RayBiotech | AAM-ANG-1-2 |
|  | Mouse Cytokine Array C1000 | RayBiotech | AAM-CYT-1000 |
| **ELISAs** | Mouse Cxcl4 ELISA | Abcam | ab202403 |
|  | Mouse FasL ELISA | RayBiotech | ELM-FasL-1 |
|  | Mouse Cxcl11 ELISA | RayBiotech | ELM-ITAC-1 |
|  | Mouse Tnfrsf11b ELISA | Abcam | ab203365 |
|  | Mouse Cxcl15 ELISA | RayBiotech | ELM-CXCL15-1 |
| **MISCELLANEOUS** | TO-PRO-3 | Thermo Fisher Scientific | R37170 |
|  | Streptavidin/Biotin Blocking Kit | Vector Labs | SP-2002 |
|  | ProLong Diamond Antifade | Thermo Fisher Scientific | P36961 |
|  | RNeasy Mini Kit | Qiagen | 74104 |
|  | QuantiTect Reverse Transcription Kit | Qiagen | 205311 |
|  | Calcein-AM | Thermo Fisher Scientific | C1430 |
|  | Buthionine Sulfoximine | Tocris Bioscience | 6954 |
|  | Carbenoxolone | Sigma-Aldrich | C4790 |
|  | DAPT | Tocris Bioscience | 2634 |
